# Influence of social determinants, diabetes knowledge, health behaviors, and glycemic control in type 2 diabetes: an analysis from real-world evidence

**DOI:** 10.1186/s12902-020-00604-6

**Published:** 2020-08-26

**Authors:** Rubén Silva-Tinoco, Teresa Cuatecontzi-Xochitiotzi, Viridiana De la Torre-Saldaña, Enrique León-García, Javier Serna-Alvarado, Arturo Orea-Tejeda, Lilia Castillo-Martínez, Juan G. Gay, David Cantú-de-León, Diddier Prada

**Affiliations:** 1 Clínica Especializada en el Manejo de la Diabetes en la Ciudad de México, Servicios de Salud Pública de la Ciudad de México, Alfonso Toro s/n, Col. Escuadrón 201, Iztapalapa, 09060 Mexico City, Mexico; 2 Servicios de Salud Pública del Gobierno de la Ciudad de México, Mexico City, Mexico; 3 Heart Failure and Respiratory Distress Clinic at Instituto Nacional de Enfermedades Respiratorias “Ismael Cosío Villegas”, Mexico City, Mexico; 4 Departamento de Nutriología Clínica, Instituto Nacional de Ciencias Médicas y Nutrición Salvador Zubirán, Mexico City, Mexico; 5 Tecnología e Información para la Salud, TIS, Mexico City, Mexico; 6 Unidad de Investigación Biomédica en Cáncer, Instituto Nacional de Cancerología - Instituto de Investigaciones Biomédicas, Universidad Nacional Autónoma de México, Mexico City, Mexico; 7 Support and Research Promotion Program (AFINES), Faculty of Medicine, Universidad Nacional Autónoma de México (UNAM), Mexico City, Mexico; 8 Department of Biomedical Informatics, Faculty of Medicine, Universidad Nacional Autónoma de México (UNAM), Mexico City, Mexico; 9 Department of Environmental Health Science, Mailman School of Public Health, Columbia University, New York City, USA

**Keywords:** Social determinants, Self-care - diabetes knowledge, Glycemic control, Physical activity, Socioeconomic status

## Abstract

**Background:**

Although important achievements have been done in type 2 diabetes mellitus (T2D) treatment and glycemic control, new strategies may take advantage of non-pharmacological approaches and of other potential determinants of health (e.g., socioeconomic status, education, diabetes knowledge, physical activity, and self-care behavior). However, the relationships between these factors are not totally clear and have not been studied in the context of large urban settings. This study aimed to explore the relationship between these determinants of glycemic control (GC) in a low-income urban population from Mexico City, focused in exploring potential the mediation of self-care behaviors in the association between diabetes knowledge and GC.

**Methods:**

A multicenter cross-sectional study was conducted in patients with type 2 diabetes (T2D) from 28 primary care outpatient centers located in Mexico City. Using multivariable-adjusted models, we determined the associations between diabetes knowledge, self-care behaviors, and GC. The mediation analyses to determine the pathways on glycemic control were done using linear regression models, where the significance of indirect effects was calculated with bootstrapping.

**Results:**

The population (*N* = 513) had a mean age of 53.8 years (standard deviation: 11.3 yrs.), and 65.9% were women. Both socioeconomic status and level of education were directly associated with diabetes knowledge. Using multivariable-adjusted linear models, we found that diabetes knowledge was associated with GC (β: -0.102, 95% Confidence Interval [95% CI] -0.189, − 0.014). Diabetes knowledge was also independently associated with self-care behavior (for physical activity: β: 0.181, 95% CI 0.088, 0.273), and self-care behavior was associated with GC (for physical activity: β: -0.112, 95% CI -0.194, − 0.029). The association between diabetes knowledge and GC was not observed after adjustment for self-care behaviors, especially physical activity (β: -0.084, 95% CI -0.182, 0.014, *p*-value: 0.062). Finally, the mediation models showed that the effect of diabetes knowledge on GC was 17% independently mediated by physical activity (*p*-value: 0.049).

**Conclusions:**

Socioeconomic and educational gradients influence diabetes knowledge among primary care patients with type 2 diabetes. Self-care activities, particularly physical activity, mediated the effect of diabetes knowledge on GC. Our results indicate that diabetes knowledge should be reinforced in low-income T2D patients, with an emphasis on the benefits physical activity has on improving GC.

## Background

Diabetes is a major worldwide cause of death and disability. It is estimated that diabetes affects 451 million adults, mainly in low- and middle-income countries (LMICs) [1, 2]. Diabetes also has a considerable financial impact on patients and health care systems [3, 4].

The document *Healthy People 2020* from the United States government has emphasized the importance of social and environmental factors that affect individuals and their health. The goal is to “reduce the disease and economic burden of diabetes mellitus and improve the quality of life for all persons who have, or are at risk for, diabetes” [5]. Historically, research and the resulting clinical approaches focusing on the individual, including pharmacological treatments, have led to improvements in self-management outcomes and reduction of cardiovascular risk factors. Despite significant advances in treatment, inadequate disease control persists [6]. However, researchers have more recently recognized the need to consider factors external to the individual, namely, social determinants of diabetes, to achieve the goal of sustainable improvement in health outcomes [7].

Although important achievements have been done in type 2 diabetes mellitus (T2D) treatment and glycemic control, new strategies may take advantage of non-pharmacological approaches and of other potential determinants of health. It is suggested that urban areas of LMICs, where the burden of disease is particularly high, need further research on this topic [8, 9]. For example, diabetes self-care activities are behaviors undertaken by T2D patients and contribute to successful self-management [10]. These self-care behaviors positively correlate with good glycemic control, reduction of complications, and improvement in the quality of life [11, 12].

According to the American Diabetes Association, the annual cost of diagnosed diabetes in America reach $327 billion, derived from more than 34 million people affected by T2D [13]. It is known that around 55% of the population lives in urban settings [14], where the burden of the disease is particularly high [15]. Only in Mexico, the prevalence of T2D in adults for 2019 reached 14% in the general population. In 2018, T2D caused 101,257 deaths in the country, making it the second leading cause of death after cardiovascular diseases [16]. There is consensus around the idea that the treatment of T2D demands changes in the model of care [17]. New therapeutic approaches suggested includes quick access to medical care, health education, motivation to carry on self-care [18–20]. The latter, in turn, demands a narrowing of the information gap between providers of care and patients [21, 22]. Evidence from several sources has shown that multidisciplinary and educational interventions yield favorable results in the control of different T2D biomarkers, improving glycemic control, compared with traditional approaches. However, this evidence derives mostly from studies in the United States and Europe, and very little is known about what is happening in low- and middle-income countries. T2D is a condition where non-pharmacological interventions on social and other determinants of health may improve health outcomes and reduce the staggering economic burden, which may derive enormous benefits in developing countries and urban areas. However, few studies have evaluated the complex interactions between social and other determinants of health in low- and middle-income countries (LMICs). Unentangling these interactions may help to focus the available resources on those more relevant factors and deriving in cost-efficient strategies.

The relationship between diabetes knowledge and health outcomes has been inconsistently reported [23–27]. However, few studies have shown the relationship between diabetes knowledge, self-care behaviors, and clinical outcomes in low-income urban populations, where diabetes knowledge could be even more inadequate. Therefore, using mediation analyses, this study aimed to explore the determinants of glycemic control (GC), particularly the mediation of self-care behaviors in the association between diabetes knowledge and glycemic control among T2D patients and low SES from Mexico City.

## Methods

### Design and population

A multicenter cross-sectional study was conducted in T2D patients from 28 primary outpatient centers located in urban areas of Mexico City. The patients were beneficiaries of Seguro Popular (public health insurance, now INSABI). Consecutive patients were invited from January 2017 to May 2018 to participate to the study through an open letter, offering free access to the program and medications. Because of the benefits in terms of high-quality medical care and guarantee of providing T2D treatment, the rates of acceptance were very high (95.0%). T2D patients who agreed to participate were referred to an outpatient diabetes center located in Iztapalapa, a municipality of Mexico City to complete an assessment. This study was registered in ClinicalTrials.gov (Identifier: NCT04245267).

### Clinical, laboratory, socio-economic, diabetes knowledge and self-care activities assessments

The data related to demographic characteristics, current treatment, time since diagnosis, comorbidities, and physical medical examination were collected from medical records and confirmed during medical interviews. Clinical and demographic data was collected based on general information that is detailed in Table 1. Clinical evaluation was done according to specialized exploration for T2D patients [28]. Data was collected by endocrinologists in a pre-designed and secured electronic form during the first interview. Endocrinologists also carried out the first clinical examination after acceptance of participation in the study. The biochemical data, including glycated hemoglobin (HbA1c) as a measure of glycemic control, were collected from the last blood tests done 2 weeks before the study. To determine the presence of diabetes-related microvascular complications, standardized criteria from international clinical practice guidelines [29] were implemented, such as fundoscopy with mydriatic camera for retinopathy assessment, increased urine albumin/creatinine ratio or decreased estimated glomerular filtration rate for diabetic kidney disease assessment, and abnormal sensitive and vibratory perception for distal diabetic neuropathy assessment [29, 30].

**Table 1:** Sociodemographic and clinical characteristics in low-income patients with type 2 diabetes mellitus in Mexico City by sex (340 females, and 173 males, *N*=513).

**Continuous variables**	**All**		**Male**		**Female**		***p*-value**
	**Mean**	**SD**	**Mean**	**SD**	**Mean**	**SD**	
Age, yrs	53.8	11.32	52.65	11.76	54.33	11.10	0.123
Body mass index, kg/m^2^	29.6	7.11	28.00	5.91	30.38	7.54	**0.003**
Years of disease	12.3	8.75	11.75	8.38	12.52	8.95	0.342
Socioeconomic status (score)^a^	80.4	40.51	85.23	40.73	77.81	40.44	0.052
Diabetes knowledge (SKILL-D Score)	3.06	2.37	3.42	2.49	2.90	2.29	**0.021**
**Self-care behavior**							
Diet							
Specific	2.90	1.85	2.81	1.77	2.94	1.91	0.452
Global	2.36	2.09	2.37	2.15	2.38	2.08	0.970
Total	2.63	1.68	2.59	1.67	2.66	1.70	0.666
Physical activity, days a week	1.85	2.31	1.88	2.20	1.83	2.38	0.819
Blood sugar testing, days a week	1.70	2.28	1.67	2.20	1.71	2.32	0.876
Foot care, days a week	3.39	3.19	3.04	3.15	3.57	3.19	0.073
Self-care global score^b^, days a week	2.37	1.57	2.29	1.43	2.41	1.64	0.371
HbA1c, %	9.64	2.19	9.45	2.24	9.74	2.16	0.166
**Categorical variables**	**n**	**%**	**n**	**%**	**n**	**%**	
**Education**							
Null	22	4.3%	8	2.4%	6	3.5%	**<0.001**
No read, no write	49	9.6%	19	5.6%	3	1.7%	
Primary school	198	38.6%	44	12.9%	5	2.9%	
Secondary school	118	23.0%	139	40.9%	59	34.1%	
Preparatory	79	15.4%	76	22.4%	42	24.3%	
University	33	6.4%	38	11.2%	41	23.7%	
No information	14	2.7%	16	4.7%	17	9.8%	
**Socioeconomic status**							
A, B (> 193)	6	1.2%	4	1.2%	2	1.2%	0.834
C+ (155 to 192)	28	5.5%	16	4.7%	12	6.9%	
C (128 to 154)	45	8.8%	31	9.1%	14	8.1%	
C- (105 to 127)	64	12.5%	40	11.8%	24	13.9%	
D+ (80 to 104)	123	24.0%	79	23.2%	44	25.4%	
D (33 to 79)	189	36.8%	127	37.4%	62	35.8%	
E (0 to 32)	20	3.9%	15	4.4%	5	2.9%	
No information	38	7.4%	28	8.2%	10	5.8%	
**Comorbidities**							
Hypertension	249	48.5%	170	50.0%	79	45.7%	0.636
High triglycerides	305	59.5%	199	58.5%	106	61.3%	0.721
High total cholesterol	248	48.3%	174	51.2%	74	42.8%	0.152
**Microvascular complications**							
Retinopathy	123	24.0%	77	22.6%	46	26.6%	0.422
Nephropathy	212	41.3%	122	35.9%	90	52.0%	**<0.001**
Neuropathy	282	55.0%	178	52.4%	104	60.1%	0.066

^a^Score for socioeconomic status. ^b^Diet, physical activity, glucose and foot care divided by 4. *SD* Standard deviation.

SES was determined using the AMAI index (Spanish for Mexican Association of Marketing Research and Public Opinion Agencies [31], which integrates updated information of income and expenses of Mexican households from official Mexican government databases. The index provides a numeric value (0 to > 193) and five categories ranging from “A/B”, the highest socioeconomic level, to “E”, the lowest [32]. Diabetes knowledge was assessed using the Spoken Knowledge in Low Literacy Patients with Diabetes (SKILLD) Scale [33]. The 10-item SKILLD assesses the knowledge of lifestyle interventions, glucose management, recognition and treatment of hypo- and hyperglycemia, and activities to prevent long-term diabetes-related complications. Each item score was summed; it ranged from 0 to 10. The higher the score number, the better the knowledge of diabetes. The SKILLD was originally designed and validated for vulnerable T2D patients with low literacy, and it has been previously used in Mexican-origin populations [34–36].

The 11-item version of the Summary of Diabetes Self-Care Activities (SDSCA) was used to measure participants’ self-care behaviors. This self-report instrument assesses participants´ frequency (over the past 7 days) of engaging in diabetes self-care behaviors, including following healthy diet, exercise/physical activity, self-monitoring of blood glucose testing, and foot care. Greater number of days indicates better self-management. Diet adherence evaluation included general diet (i.e., days a week patients followed healthy diet), specific diet (i.e., days a week patient eat adequate portions of fruits and vegetables/and avoid fat-rich food), and global diet (i.e., average obtained from the general and specific diet) [37].

Even though patients were referred from different centers, HbA1c was determined in a centralized laboratory using immunoassays National Glycohemoglobin Standardization Program (NGSP)-certified methods [38]. In particular, we used tubidimetric inhibition immunoassay for HbA1c (CobasC-111, Roche Diagnostics International Ltd., USA).

### Statistical analyses

**Fig. 1:**
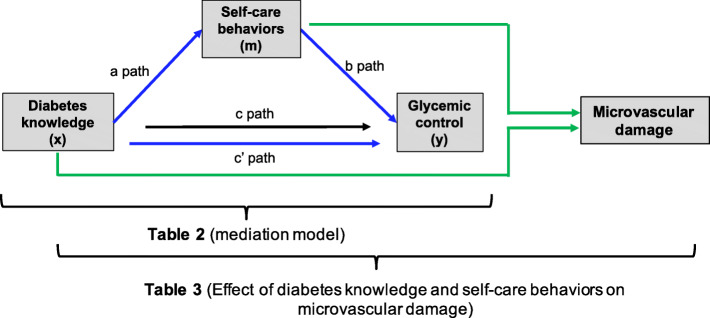
Conceptual models used in this study and results shown in the corresponding tables. Blue arrows: Single mediator models for diabetes knowledge effect on glycemic control (y) via behavioral determinants (m). Path a represents the association between diabetes knowledge (x) and individual behavioral determinants (m). Path b represents the relation between individual behavior determinants (m) and glycemic control (y). c path represents the crude association between diabetes knowledge (x) and glycemic control (y). c’ path represents the association between diabetes knowledge (x) and glycemic control (y) corrected for behavioral determinant (m). Green arrows: Effect of diabetes knowledge and self-care behaviors on microvascular damage

We examined the role of diabetes knowledge in self-care behavior; we used univariable and multivariable linear models adjusted for age, sex, and time since diagnosis. We also used multiplicative terms to determine the potential interactions between socioeconomic status (SES) in the association between diabetes knowledge and self-care behaviors. We determined the role of education in the association between SES and diabetes knowledge using multivariable logistic regression models. We evaluated the role of self-care behaviors in the association between diabetes knowledge and glycemic control, including diet (specific, global, and total), physical activity, glucometry assessments, foot care, and a self-care global score. We ran mediation models according to the Baron and Kenny’s steps [39]. We assessed whether the effect of diabetes knowledge on glycemic control (HbA1C levels) was mediated by self-care behaviors in a multiple mediator model (Fig. 1, Additional File 1). Coefficients were obtained from a linear regression analysis. Indirect effects were calculated based on the product-of-coefficients method (a*b) [40]. Standard errors and confidence intervals for mediation analyses were calculated with bootstrapping (5000 samples) [41]. Outcome and mediating variables were adjusted for age (as a continuous variable), sex (male vs. female), years of disease (as a continuous variable), and education level (primary vs. higher education). The direct effect (c’ path) did not have to be reduced to zero because an incomplete mediation of the effect was expected. Also, for significant mediating variables, the proportion mediated was calculated as effect size measure ((a*b)/c). Finally, we determined the role of diabetes knowledge and self-care behaviors in glycemic control (HbA1c levels, as a continuous variable) and microvascular damage (retinopathy, nephropathy, and neuropathy) using multivariable-adjusted logistic regression models. We performed the analyses using the R software (R Project for Statistical Computing, CRAN, The Comprehensive R Archive Network, Vienna) and we used the command mediate() in ‘mediation’ package for bootstrapping. Statistical significance was defined as a value of *p* < 0.05.

## Results

### Characteristics of study participants

A total of 513 T2D patients were included, of which 66.3% were female. The mean age was 53.7 years (standard deviation [SD] 11.32 years). Most of the population reported primary school or less as their education level (52.4%), and 64% showed low or very low socioeconomic status (D+, D, and E, AMAI categories). Patients had a mean time since diagnosis of 12.2 years (SD: 8.75 years), and 48.4% of them reported coexisting hypertension. The microvascular complications assessment revealed the presence of diabetic retinopathy in 23.9% of the population, diabetic renal disease in 41.3%, and diabetic distal neuropathy in 54.9%. The SKILLD scale, used to measure diabetes knowledge, showed a mean value of 3.06 (SD: 2.37, range 0–10). The best self-care activity performance observed was foot care (mean 3.39 days a week, SD: 3.19 days a week), and glucose blood sugar testing was the least commonly performed (mean 1.7, SD: 2.28 days a week). The mean level of HbA1c was 9.6% (SD: 2.2). Full description of the population included is shown in Table 1.

### Role of socioeconomic factors in diabetes knowledge and sex-effects

Sex-effects were observed, with significant associations for higher diabetes knowledge, higher education level, and nephropathy for women (Table 1, Additional File 2). We found that SES was linked to education level (*p*-value < 0.001, Supplementary Fig. 1, Additional File 3). Multivariable-adjusted models also showed that SES was associated with diabetes knowledge (β: 0.009, 95% Confidence Interval [95% CI] 0.003, 0.015, *p*-value < 0.001, Supplementary Table 1, Additional File 3). Finally, univariable and multivariable-adjusted models showed a positive and statistically significant association between education and on diabetes knowledge. Education showed a strongest effect than SES and was significant on both males and females. SES was associated with diabetes knowledge only in female participants (Supplementary Table 1, Additional File 3).

### Mediation analysis of self-care activities in the association between diabetes knowledge and glycemic control

Table 2 (Additional File 4) shows the mediation of self-care behaviors between diabetes knowledge and glycemic control. First, we determined the effect of diabetes knowledge on glycemic control (c path). Multivariable regression models, adjusted for age, sex, years of disease, and education, showed a negative association between diabetes knowledge and glycemic control (HbA1C blood levels, *p*-value = 0.023). Second, we examined the impact of diabetes knowledge on self-care behaviors using multivariable- adjusted linear models (a path). Diabetes knowledge was significantly associated with all self-care behaviors, including diet (general, specific, and global score), physical activity, blood sugar testing, and foot care, and with the global score for self-care activities. The strongest association was observed for foot care and the weakest for specific diet score. Following the Baron and Kenny’s steps for mediation models [39], we determined the role of self-care behaviors in glycemic control (b path). Among self-care behaviors, only physical activity and global diet reached statistical significance. The association between diabetes knowledge and glycemic control was lost after adjustment for self-care behaviors, especially physical activity. Finally, we determined if the mediation effect was statistically significant. We found a partial mediation of physical activity in the association between diabetes knowledge and glycemic control (Table 2, Additional File 4), where physical activity significantly accounted for 17% of the association (*p*-value: 0.049). We evaluated if the mediation was sex-specific, but although the direction of the association was still present, we did not observe statistical significance.

**Table 2:** Mediation of self-care behaviors between diabetes knowledge and glycemic control in low-income patients with type 2 diabetes mellitus from Mexico City (*n*=513).

**Diabetes knowledge**	X → Y (c path)^a,h^												
	**β**	**95% CI**											
Crude analysis	-0.10	(-0.19, -0.01)											
	X → Y (c' path) direct^b,h^		X → M (a path)^c,h^		M → Y (b path)^d,h^			Indirect effect (a*b)^e,g^			Proportion mediated^f^		
	**β**	**95% CI**	**β**	**95% CI**	**β**	**95% CI**		**β**	**95% CI**		**β**	**95% CI**	
**Self-care behavior**													
Diet												(-0.30, 0.87)	
Specific	-0.11	(-0.02, -0.19)	0.13	(0.06, 0.21)	0.06	(-0.04, 0.17)		0.01	(0.00, 0.03)		-0.11	(-0.64, 0.04)	
Global	-0.09	(-0.17, 0.02)	0.18	(0.09, 0.26)	-0.09	(-0.10, 0.00)		-0.01	(-0.03, 0.00)		0.13	(-0.05, 0.75)	
Total	-0.10	(-0.12, 0.11)	0.15	(0.09, 0.22)	-0.03	(-0.15, 0.08)		0.00	(-0.02, 0.02)		0.01	(-0.27, 0.34)	
Physical activity, days a week	-0.08	(-0.18, 0.01)	0.18	(0.09, 0.27)	-0.11	(-0.19, -0.03)		-0.02	(-0.04, 0.00)		0.17	(0.01, 0.85)	
Blood sugar testing, days a week	-0.10	(-0.06, 0.12)	0.13	(0.04, 0.22)	0.02	(-0.07, 0.10)		0.00	(-0.01, 0.02)		-0.04	(-0.43, 0.19)	
Foot care, days a week	-0.09	(-0.07, 0.06)	0.40	(0.28, 0.53)	-0.02	(-0.08, 0.04)		0.00	(-0.03, 0.02)		0.02	(-0.60, 0.49)	
Self-care global score, days a week	-0.09	(-0.19, 0.07)	0.23	(0.17, 0.28)	-0.10	(-0.22, 0.03)		-0.01	(-0.05, 0.02)		0.13	(-0.30, 0.87)	

^a^ c path (total effect): The crude association between diabetes knowledge and glycemic control.

^b^ c' path (direct effect): the association between diabetes knowledge and glycemic control, adjusted for mediator (self-care behavior)

^c^ a path: association between diabetes knowledge and self-care behavior

^d^ b path: association between self-care behavior and glycemic control

^e^ Indirect effect (a*b): the indirect effect of the diabetes knowledge on glycemic control through self-care behavior

^f^ Proportion effect mediated ((a*b)/c): the proportion of the total effect mediated through self-care behavior

^g^ Confidence interval for indirect effects were calculated with bootstrapping (5000 samples)

^h^ All analyses used linear regression models adjusted for age, sex, years since diagnosis, and SES

### Role of diabetes knowledge and self-care in microvascular damage

We studied whether diabetes knowledge or self-care behaviors were associated with microvascular damage (retinopathy, nephropathy, and neuropathy). Diabetes knowledge was not associated with any of the surrogates for microvascular damage. However, several self-care behaviors were associated with neuropathy, including diet (general HR: 0.95, 95% CI 0.90, 0.99; global HR: 0.95, 95% CI 0.92, 0.99), physical activity (HR: 0.90, 95% CI 0.83, 0.98), blood sugar testing (HR: 0.86, 95% CI 0.79, 0.93), and self-care global score (HR: 0.97, 95% CI 0.95, 0.99) (Table 3). Global diet score was also associated with retinopathy (HR: 1.04, 95% CI 1.01, 1.09), and physical activity was associated with the presence of any of the surrogates of microvascular damage (HR: 0.83, 95% CI 0.72, 0.96, Table 3, Additional File 5).

**Table 3:** Association Among Diabetes Knowledge and Self-Care Activities on Microvascular Complications in low-income patients with type 2 diabetes mellitus in Mexico City (*n*=513).

	**Retinopathy**		**Nephropathy**		**Neuropathy**		**Any surrogate for microvascular damage**	
**Variable**	**β**	***p*-value**	**β**	***p*-value**	**β**	***p*-value**	**β**	***p*-value**
	**95%CI**		**95%CI**		**95%CI**		**95%CI**	
**Knowledge in diabetes**	-0.06	0.324	-0.01	0.837	-0.02	0.638	-0.02	0.755
	(-0.17, 0.06)		(-0.09, 0.07)		(-0.10, 0.06)		(-0.14, 0.10)	
**Diet**								
Specific	0.07	0.073	0.00	0.911	-0.06	0.050	0.00	0.957
	(-0.01, 0.14)		(-0.06, 0.05)		(-012. 0.00)		(-0.08, 0.74)	
Global	0.05	0.075	0.04	0.095	-0.06	**0.017**	0.01	0.744
	(-0.01, 0.11)		(-0.01, 0.08)		(-0.10, -0.01)		(-0.06, 0.08)	
Total	0.04	**0.038**	0.01	0.373	-0.05	**0.005**	0.00	0.860
	(0.01, 0.08)		(-0.02, 0.05)		(-0.08, -0.02)		(-0.04, 0.05)	
**Physical activity**	-0.05	0.399	0.01	0.883	-0.10	**0.012**	-0.19	**0.009**
	(-0.16, 0.06)		(-0.07, 0.08)		(-0.19, -0.02)		(-0.33, -0.05)	
**Blood sugar testing**	-0.01	0.851	0.00	0.989	-0.15	**< 0.001**	-0.12	0.082
	(-0.09, 0.10)		(-0.08, 0.08)		(-0.23, -0.07)		(-0.26, 0.02)	
**Foot care (score)**	-0.01	0.815	0.00	0.970	-0.04	0.226	-0.05	0.277
	(-0.09, 0.07)		(-0.06, 0.06)		(-0.10, 0.02)		(-0.14, 0.04)	
Self-care global score**	0.02	0.210	0.01	0.249	-0.03	**0.001**	-0.06	0.263
	(-0.01, 0.04)		(-0.01, 0.03)		(-0.05, -0.01)		(-0.18, 0.05)	

*Socioeconomic status score included as continuous variable. Models adjusted by age (continuous), gender (categorical) and years of disease (continuous). 95% CI: 95% Confidence interval.

## Discussion

This study showed how diabetes knowledge plays a key role on diabetes outcomes through self-care behaviors. Remarkably, our study found that, among all the self-care behaviors evaluated, physical activity mediated the association between diabetes knowledge and glycemic control in a low-income population of T2D patients from a large urban area. Both SES and level of education were directly associated with diabetes knowledge, but education showed a stronger impact. Additionally, we found a decreased risk for developing microvascular diabetes-related complications (particularly with distal diabetic neuropathy), with a higher self-care score (particularly with physical activity). To the best of our knowledge, this is the first deep exploration of determinants of health in population with T2D living in a large populated area in Mexico City.

Some previous studies in T2D have determined the mediation of behavioral determinants on the effectiveness of lifestyle interventions in changing behavior and body weight. Texeira et al. found that motivation, self-efficacy, and self-regulation skills were reported as mediators of weight change and physical activity behavior [42]. Den Braver et al. also reported that SLIMMER (SLIM iMplementation Experience Region Noord- en Oost-Gelderland) intervention on fasting insulin and body weight was mediated by changes in dietary and physical activity behavior [43]. Mediation models identify and explain the process that underlies an observed relationship between an independent variable (diabetes knowledge) and a dependent variable (glycemic control) via the inclusion of a third hypothetical variable, known as a mediator variable (i.e., self-care behaviors. To our knowledge, no previous study has determined the potential mediation of self-care behaviors in population from Mexico City, which is particularly susceptible to abnormal cardiometabolism and the worst prognosis [44]. For mediation analysis, we used one of the two available approaches: the Sobel test and bootstrapping [41]. Although the Sobel test has been widely used since 1982, bootstrapping has been strongly recommended in recent years. Hence, we chose to bootstrap our mediation analysis. Using this approach, we determined that physical activity mediated the association between diabetes knowledge and glycemic control in this population setting. This finding coincides with those reported by den Braver et al. [43], and allows us to focus our non-pharmacologic interventions of this self-care behavior. As the American Association of Diabetes has insisted, physical activity is critical for blood glucose management and overall health in individuals with diabetes [45], and we ask on its importance in LMICs.

Our population showed some significant differences related to sex-effects. Notably, we found that diabetes knowledge scores were higher in males; however, the education level was higher in women than in men. Also, a higher frequency of neuropathy was observed in women. Sex differences in the impact of T2D on CVD outcomes across the life span have been previously identified [46, 47]. For example, T2D confers 25–50% greater excess risk of incident cardiovascular disease in women compared with men. Obesity trends in women may, in part, explain the observed sex difference in T2D in midlife. In fact, we observed a higher and significant body mass index in females. A deep analysis of sex-dependent differences and the relevance of the observed outcomes are guaranteed.

The present study is in line with previous studies about the benefit of self-care behaviors, particularly physical activity, and provides additional information on the causal path. This study also shows the relevance of diabetes knowledge and self-care behavior in T2D patients. The lack of health-related knowledge and poor performance of diabetes self-care habits could partially explain the heavier diabetes burden in populations with social lag indicators. Previously, diabetes was associated with worse prognoses in Mexico than in high-income countries [44, 48] which made this research obligatory.

Our results showed that self-care behaviors were linked to microvascular damage in any form, with deeper reductions in Hazard Ratios for physical activity and blood sugar testing. Previous studies have also found similar results, both cross-sectionally as longitudinally [49, 50]. However, our study was collected based on self-report data at the time of the interview, and cross-sectionally analyzed. Therefore, because of the nature of the analysis, our results cannot suggest that self-care behavior will derive in reductions in microvascular complications. The effect of interventions and improvements in self-care behavior in T2D patients on long-term microvascular complications in LMICs to establish causality is guaranteed. We also observed some unexplained associations (e.g., global diet and retinopathy), that we could not explain and that could be due to potential confounders not included in our statistical models.

An important finding was the elucidation of socioeconomic factors on diabetes knowledge. Both factors evaluated, SES and education level, played a major role in diabetes knowledge, particularly education. This finding suggests there is a causative role of socioeconomic factors in the epidemic of complications of diabetes mellitus in underdeveloped countries. Poverty influences the development of type 2 diabetes and complications [51]. Education and socioeconomic levels are associated with the activation of self-care management in chronic diseases [52]. These two social determinants could partially influence the poor performance in self-care habits, through the potential contribution and mediating effect of poor diabetes knowledge, which indicates a link between social determinants of health and diabetes self-management. Moreover, socioeconomic status was directly associated with glucose testing, which identifies the restriction T2D patients and low SES have to execute this behavior. These findings reiterate why health care professionals need to consider their patients’ socioeconomic status when implementing diabetes self-care management and education programs.

One strength of this study is that it included a validated tool for diabetes knowledge (SKILLD), which was designed for vulnerable populations. We observed that most patients scored low on this scale, even though T2D patients with long-term diabetes diagnosis were enrolled, which indicates the need for increasing diabetes education in healthcare programs. The study participants were beneficiaries of Seguro Popular (now INSABI) in Mexico City, which attends to the largest number of primary care outpatients in Mexico. Therefore, our results were obtained from real-world data among a representative population from a low-income subset of patients from one of the largest urban areas in the world, which is home to more than 20 million people in 1450 km^2^.

Although our results are novel and potentially useful in the context of diabetes mellitus treatment in low-income countries, our study has several limitations. First, this is a cross-sectional epidemiological study, so we are not able to conclude causality. However, as any other epidemiological study, it is a hypothesis generator, and examines the relevance of education on diabetes knowledge to improve glycemic control. Second, the sample size may seem small in comparison with other diabetes studies. However, our study included a large number of participating health centers in an unprecedented manner. Third, although this study was conducted in Mexico City, a megalopolis, our findings may not be representative of other urban areas across the world. Nevertheless, the study highlights the need to consider social determinants of health and diabetes knowledge across populations that surely share similarities with our sample. Fourth, the SKILLD scale does not have a widespread use among studies that measure diabetes knowledge. Moreover, from our perspective, the traditional tools used for this purpose are difficult to understand for populations with educational lag; hence we decided to administer SKILLD, which has been tested and validated in populations similar to ours. Fifth, despite adjusting for potential confounding factors in our analysis, we did not assess for other factors that may influence glycemic control and self-care behaviors, such as pharmacological treatments, mental disorders, barriers to self-care, etc. Adverse diabetes outcomes are complex and multicausal and involve biological, individual, and social factors. In this work, we try to reinforce the relevance of some of them; we recognize the difficulty of including all the factors involved in a study of this nature.

We also recognize that our study did not evaluate the frequency of smoking in the participants. However, this analysis is part of an interventional program in which the educational component includes strict recommendations to stop smoking and to avoid second-hand smoking to all participants, even though this factor is not present [27]. Future studies about the role of smoking in LMICs are guaranteed. As the inclusion to the study was not randomized and our study was not controlled due to ethical concerns, our results might be affected by selection bias. Only those participants who accepted were included in the program, and they usually lived in areas close to the clinic (i.e., mobility in Mexico City tend to be very complicated on daily basis, and people usually spend hours to move from home to workplaces). However, characteristics of the population shown in Table 1 suggested representativity of the population living at the center of the country in terms of SES, education, diet, and body mass index [53]. The success of the program will allow us to include and evaluate a higher number of participants to determine the generalizability of our results to LMICs. Finally, we recognize that usage of self-reported data may be less reliable because of the potential lack of accuracy of responses, as well as potential intentional and unintentional reporting bias. However, some studies have found that the self-report responses may be 100% in agreement with the direct observations held, suggesting that self-report methodologies can confidently be used in instances where observation may not be feasible. Therefore, our results have to be analyzed in the context of these potential biases for self-reported data.

## Conclusions

In conclusion, our study revealed that socioeconomic and educational gradients influence diabetes knowledge among primary care patients with type 2 diabetes. We also determined that self-care behaviors, particularly physical activity, mediate the effect of diabetes knowledge on glycemic control. These results may indicate the most relevant pathways to consider in populations with poor access to diabetes care, which could lead to allocating government resources to improve education and diabetes knowledge and to encouraging T2D patients to increase self-care activities, particularly physical activity. Further research is needed to estimate the size effect of interventions on diabetes knowledge and self-care improvement strategies in socially disadvantaged circumstances, particularly in low-income groups.

## Supplementary information

**Additional file 1: Supplementary material.** It includes a detailed description for the association between education and socioeconomic status on diabetes knowledge, and a supplementary figure about socioeconomic score by education level in patients with type 2 diabetes mellitus.

## Data Availability

The datasets generated and/or analyzed during the current study are not publicly available due that data used are from patients from the Clinic and are under policies for private confidential information, but are available from the corresponding author on reasonable request and using unidentifiable IDs by a third party.

## Abbreviations

GC: Glycemic control
T2D: Type 2 diabetes
LMICs: Low- and middle-income countries
SES: Socioeconomic status
HbA1c: Glycated hemoglobin
SKILLD: Spoken knowledge in low literacy diabetes
