# Supplementary material for: Influence of social determinants, diabetes knowledge, health behaviors, and glycemic control in type 2 diabetes: an analysis from real-world evidence
Source: BMC Endocr Disord. 2020 Aug 26;20:130. doi: 10.1186/s12902-020-00604-6 (PMC7449009; doi:10.1186/s12902-020-00604-6)
Supplement: Supplementary file 1 — Additional file 1: Supplementary material. It includes a detailed description for the association between education and socioeconomic status on diabetes knowledge, and a supplementary figure about socioeconomic score by education level in patients with type 2 diabetes mellitus. [file 12902_2020_604_MOESM1_ESM.docx]

**Supplementary material**

**Determinants of Glycemic Control among Urban Patients with Type 2 Diabetes and Low Socioeconomic Status**

Rubén Silva-Tinoco Rubén, Teresa Cuatecontzi-Xochitiotzi, Viridiana De la Torre-Saldaña, Enrique León-García, Javier Serna-Alvarado, Arturo Orea-Tejeda, Lilia Castillo-Martínez, Juan G. Gay, David Cantú-de-León, Diddier Prada.

SUPPLEMENTARY TABLES

| **Supplementary Table 1**. Association between education and socioeconomic status on diabetes knowledge (SKILL-D Score) in low-income patients with type 2 diabetes mellitus in Mexico City (n=513). | | | | | | | | |
| --- | --- | --- | --- | --- | --- | --- | --- | --- |
| **Variable** |  | **Univariable^a^** | | |  | **Multivariable^b^** | | |
|  |  | **β** | **95%CI** | ***p*-value** |  | **β** | **95%CI** | ***p*-value** |
| Education | All | 1.640 | (1.250, 2.036) | **< 0.001** |  | 1.533 | (1.122, 1.945) | **< 0.001** |
|  | Males | 1.259 | (0.505, 2.012) | **0.001** |  | 1.274 | (0.455, 2.094) | **0.003** |
|  | Females | 1.777 | (1.308, 2.246) | **< 0.001** |  | 1.490 | (0.965, 2.015) | **< 0.001** |
| SES | All | 0.010 | (0.005, 0.015) | **< 0.001** |  | 0.010 | (0.005, 0.015) | **< 0.001** |
|  | Males | 0.010 | (-0.002, 0.016) | 0.119 |  | 0.040 | (-0.05, 0.014) | 0.381 |
|  | Females | 0.010 | (0.004, 0.016) | **0.001** |  | 0.007 | (0.001, 0.013) | **0.029** |
| a. Based on a simple linear regression model considering diabetes knowledge (in continuous) as dependent variable and education (dichotomized, primary or lower vs. secondary or higher) or SES as the covariate of interest. b. Based on a multivariable linear regression model considering diabetes knowledge (in continuous) as dependent variable, education (dichotomized, primary or lower vs. secondary or higher) or SES as the covariate of interest and age and gender (only for the full group) as covariates. SES: Socioeconomic status. | | | | | | | | |

**SUPPLEMENTARY FIGURES**

**Supplementary Figure 1**. Socioeconomic score by education level in patients with type 2 diabetes mellitus in Mexico City (n=513). **p*-value < 0.01; ***p*-value < 0.001, Kruskal-Wallis test.
